# Fabrication and Evaluation of Voriconazole Loaded Transethosomal Gel for Enhanced Antifungal and Antileishmanial Activity

**DOI:** 10.3390/molecules27103347

**Published:** 2022-05-23

**Authors:** Mudassir Farooq, Faisal Usman, Sumera Zaib, Hamid Saeed Shah, Qazi Adnan Jamil, Fatima Akbar Sheikh, Ajmal Khan, Sameh Rabea, Soheir A. A. Hagras, Gaber El-Saber Batiha, Imtiaz Khan

**Affiliations:** 1 Department of Pharmaceutics, Faculty of Pharmacy, Bahauddin Zakariya University, Multan 66000, Pakistan; mudassirfaroooq@gmail.com; 2 Department of Biochemistry, Faculty of Life Sciences, University of Central Punjab, Lahore 54590, Pakistan; sumera.zaib@ucp.edu.pk; 3 Institute of Pharmaceutical Sciences, University of Veterinary and Animal Sciences, Lahore 54000, Pakistan; 4 Department of Pharmaceutics, Faculty of Pharmacy, The Islamia University of Bahawapur, Bahawalpur 66000, Pakistan; qazi.adnan@iub.edu.pk; 5 Al-Raziq College of Pharmacy, Sargodha 40100, Pakistan; fatimatahir303@gmail.com; 6 Natural and Medical Sciences Research Center, University of Nizwa, Nizwa 616, Oman; ajmalkhan@unizwa.edu.om; 7 Department of Pharmaceutical Sciences, College of Pharmacy, AlMaarefa University, Diriyah, Riyadh 13713, Saudi Arabia; srabea@mcst.edu.sa; 8 Department of Clinical Laboratory Sciences, Inaya Medical Colleges, Riyadh 11352, Saudi Arabia; soheirhagras@gmail.com; 9 Department of Drug Radiation Research, National Center for Radiation Research and Technology (NCRRT), Egyptian Atomic Energy Authority, Cairo 11787, Cairo, Egypt; 10 Department of Pharmacology and Therapeutics, Faculty of Veterinary Medicine, Damanhour University, Damanhour 22511, Albeheira, Egypt; gaberbatiha@gmail.com; 11 Department of Chemistry and Manchester Institute of Biotechnology, The University of Manchester, 131 Princess Street, Manchester M1 7DN, UK

**Keywords:** fungal infection, leishmaniasis, phospholipids, transethosomes, transethosomal gel, voriconazole

## Abstract

Voriconazole (VRC) is a broad-spectrum antifungal agent belonging to BCS class II (biopharmaceutical classification system). Despite many efforts to enhance its solubility, this primary issue still remains challenging for formulation scientists. Transethosomes (TELs) are one of the potential innovative nano-carriers for improving the solubility and permeation of poorly soluble and permeable drugs. We herein report voriconazole-loaded transethosomes (VRCT) fabricated by the cold method and followed by their incorporation into carbopol 940 as a gel. The prepared VRCT were evaluated for % yield, % entrapment efficiency (EE), surface morphology, possible chemical interaction, particle size, zeta potential, and polydispersity index (PDI). The optimized formulation had a particle size of 228.2 nm, a zeta potential of −26.5 mV, and a PDI of 0.45 with enhanced % EE. Rheology, spreadability, extrudability, in vitro release, skin permeation, molecular docking, antifungal, and antileishmanial activity were also assessed for VRCT and VRC loaded transethosomal gel (VTEG). Ex-vivo permeation using rat skin depicted a transdermal flux of 22.8 µg/cm^2^/h with enhanced efficiency up to 4-fold. A two-fold reduction in inhibitory as well as fungicidal concentration was observed against various fungal strains by VRCT and VTEG besides similar results against L-donovani. The development of transethosomal formulation can serve as an efficient drug delivery system through a topical route with enhanced efficacy and better patient compliance.

## 1. Introduction

Fungal and *Leishmania* infections are life-threatening to the entire population, especially in immune-compromised patients in underdeveloped countries. The mortality rate is still high owing to the development of microbial resistance, poor diagnosis, and the limitations of therapy [1,2]. *Candidiasis* and *Aspergillosis* are the most common fungal infections ranging from mucosal and superficial infections to invasive tissue infections. *Candida* infects the mouth, nail, skin, foot, and bloodstream, whereas *Aspergillus* infects the skin, hair, eyes, and lungs [3,4]. *Leishmania* develops a severe infection of the liver, spleen, and mucocutaneous tissue [5]. Recent studies on fungal and leishmaniasis diseases have demonstrated excellent voriconazole (VRC) efficacy against *Candida*, *Aspergillus*, and *Leishmania* species [6]. Currently, VRC is available for systemic delivery via oral and IV formulation only. VRC is a BCS class-II drug with low solubility and high permeability. Renal impairment occurs due to the systemic administration of VRC. Various efforts have been carried out to enhance its solubility, however, significant progress was stalled due to less control, instant release, and less stability, which eventually enhanced the formulations’ complications. The strategy of pharmaceutical innovation has been exploited for enhancing solubility and increased activity to treat fungal and *Leishmania* infection locally. Conventional topical delivery decreases skin permeability and causes skin irritation. A conventional lipid-based vesicle system, “liposomes” are not proficient enough to cross the stratum corneum and accumulate on the upper layer of the stratum corneum because of their low permeability into the deeper layer [7].

Liposomes are added with edge activators and permeation enhancers to form transethosomes (TELs) to permeate the drug via the stratum corneum. It has been observed that drugs entrapped in TELs are released in a controlled manner. TELs have a high entrapment efficiency due to the biodegradable and biocompatible nature of surfactants. TELs can entrap both low and high molecular weight drugs. Phospholipids and surfactants are used as a carrier for delivering drugs via the skin. They can easily interact with the stratum corneum, improve tissue hydration, and merge with lipids of the stratum corneum. They contain a hydrophilic (polar) head and a hydrophobic (nonpolar) tail. Biocompatible surfactant is a bilayer softening agent that increases flexibility and permeability. When alcohol and the surfactant are combined, it leads to a rearrangement of the lipid bilayer and becomes more deformable such that the deeper penetration into the dermis becomes more feasible. Furthermore, TELs can be transformed into a gel for ease of topical application. The TELs are prepared by the mechanical dispersion technique and the hot and cold method. The mechanical dispersion technique can be utilized by mixing chloroform and ethanol in a round bottom flask followed by the addition of phospholipid. The solvent is removed by using a rotary evaporator. The thin lipid film is deposited on the top of a round bottom flask and then the thin film is hydrated. In the hot method, phospholipids are dispersed in water at 40 °C until a colloidal solution is formed. In the second step, ethanol, surfactant, cholesterol, and propylene glycol are mixed in another vessel at 40 °C. Finally, the organic phase is added to the liquid phase and stirred for 10 min. The formulation is dried by lyophilization. In the cold method, the phospholipid, cholesterol, surfactant, and drug are added to ethanol, followed by the addition of propylene glycol. The aqueous phase is added dropwise at 30 °C and stirred and homogenized to obtain transethosomal dispersion. The transethosomal gel can be prepared by adding transethosomal dispersion in a foe prepared gel of carbopol 940 to enhance the permeation as well as efficacy at the target site [7,8,9].

In alignment with this perspective, the present study was designed to fabricate transethosomes using various lipids. This was followed by their incorporation into a gel form to evaluate the antifungal as well antileishmanial activity by carrying out the various formulations’ aspects. The formulations were characterized as well as molecular docking, antifungal and antileishmanial activity were performed to provide a detailed picture of targeted therapy.

## 2. Results

Voriconazole-loaded transethosomal formulations (TE1-TE8) were prepared by varying phospholipid and ethanol concentrations. These transethosomal formulations were transformed into a gel (TEG1-TEG) by using carbopol 940. Its physio-chemical parameters were also evaluated.

### 2.1. Zeta Potential, Particle Size, and Polydispersity Index

VRCT showed a vesicular size from 133.9 to 342.5 nm. TE2 and TE6 showed vesicles of 229.8 and 228.2 nm in size with a narrow size distribution (PDI) of 0.41 and0.450. PDI values lower than 0.5 indicate a monodisperse system and vesicular systems with a PDI < 0.5 meet the necessary conditions as required for the pharmaceutical’s delivery. Similar results were reported by Khan, 2020, in which they prepared rifampicin-loaded nisosomes. It is evident that vesicles with a PDI of less than 0.5 are considered to have no or less aggregation with a low polydispersity indicating a stable formulation system [10,11]. Similar reports are mentioned by Pereira-Lachataignerais, 2006 where accordingly, vesicular formulations with a PDI in the range of 0.23 to 0.51 are indicative of relatively homogeneous dispersion [12].

The TE2, TE4, TE6, and TE8 containing Span 60 showed negative zeta potential, following stable formulation as reported by Mahmoud H. Teaima, 2020. TE1, TE3, TE5, TE7 containing Tween 80 showed neutral zeta potential [13]. The zeta potential lies in the range of 25–32 mV, showing that particles are monodispersed. Negative and Positive zeta potential values are related to various emulsifiers used in the formulations. The presence of the charge may be advantageous in maintaining the formulation’s stability as electrostatic repulsive forces reduce the chances of particle aggregation. It has also been reported that a mild surface charge may be related to the presence of impurities in the lipids [14,15]. The results of zeta potential, particle size, and polydispersity index are shown in Table 1.

### 2.2. Fourier-Transform Infrared Spectroscopy (FT-IR)

**Figure 1:**
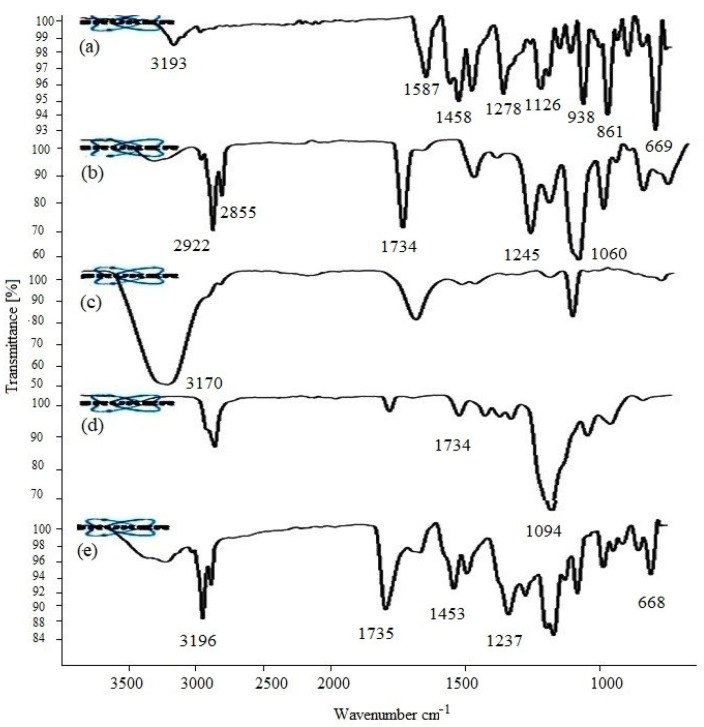
FTIR spectra of pure voriconazole (**a**), lipoid S100 (**b**), carbopol (**c**), Tween 80 (**d**), and voriconazole, carbopol, and lipoid S100 mixture (**e**).

The spectral bands of pure drug, carbopol 940, phospholipid, Tween 80, and VRC, carbopol–lipoid S100 mixture are shown in Figure 1. VRC depicted distinguishing absorption peaks showing strong aromatic bending at 958, 861, 669 cm^−1^; C-N stretching appeared at 1278 cm^−1^; and C-C at 1587 cm^−1^. The fluorine bands displayed at 1008 cm^−1^, C = C at 1458 cm^−1^, and C-O stretching at 1126 cm^−1^. VRC showed weak OH stretching at 3193 cm^−1^. Lipoid S100 showed distinguished P-O-C stretching at 1060 cm^−1^, P = O stretching at 1245 cm^−1^, and 1734 cm^−1^ stretching due to the presence of an ester group. The bands observed at 2922 and 2855 cm^−1^ were due to the stretching vibration of CH_2_ and CH_3_ groups as shown in Figure 1. Carbopol 940 showed typical intermolecular hydrogen bonds at 3170 cm^−1^ (bonded hydroxyl) and at 3526 cm^−1^ (free hydroxyl). Tween 80 bands appearing at 1734 and 1094 cm^−1^ are assigned to the stretching vibration of C = O and C-O-C, respectively.

### 2.3. Optical Microscopy

**Figure 2:**
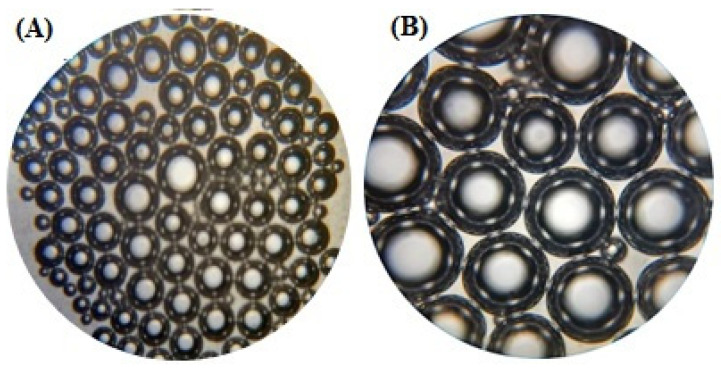
Optical microscopy of TE6 at 10× (**A**) and 40× (**B**) magnification.

An optical microscope confirmed the formation of spherical vesicles. TELs were uniformly distributed and found to be somewhat spherical in shape. Optical images at a high magnification demonstrated the inner structure of the prepared lipid vesicles. A hybrid-nanostructured dense lipid vesicle composing an outer phospholipid layer with an empty core was observed, as depicted in Figure 2.

### 2.4. Scanning Electron Microscopy (SEM)

**Figure 3:**
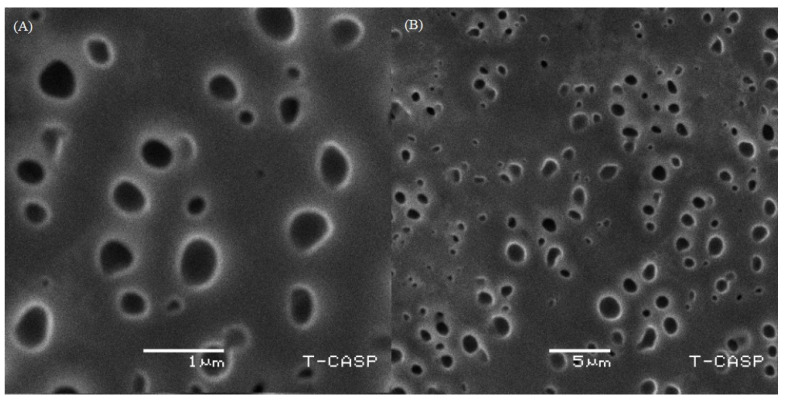
SEM of TE6 formulation at 1µm (**A**) and 5 µm (**B**).

The structure of the optimized TEG6 formulation was examined by SEM at a resolution of 1 and 15 µm as shown in Figure 3. The spherical-shaped vesicles were dominant in the formulation while irregular shapes were limited. It was observed that SEM images of the optimized formulation contain pores which facilitate the adherence of the drug into the interpenetrating network of the TELs. Figure 3 shows the dispersed white particles at a high magnification throughout the network which refers to the model drug loaded in TELs.

### 2.5. % Entrapment Efficiency

The % EE of TELs was 73 to 86%. TE6 showed 86% % EE containing 35% ethanol and 3% lipoid S100. TE4, TE3, TE7 and TE6 showed minimal EE owing to the use of lipoid SP-C3. TE1 and TE2 also had a lower % EE because of a decreasing ethanol concentration of up to 30%.

### 2.6. Storage Stability of Transethosomes

The stability study revealed that TE3, TE4, TE7, and TE8 were not stable owing to a decrease in % EE, a slight change in pH, and an increase in particle size due to the aggregation of vesicles. The formulations TE1, TE2, TE5, and TE6 with lipoid S100 were stable, only showing minor changes in particle size, PDI, and % EE as shown in Table 2.

### 2.7. Transethosomal Gel

#### 2.7.1. Appearance, pH, and Conductivity

The transethosomal gel of all formulations was smooth, homogeneous, and milky in appearance. The pH of transethosomal gel was found in the range of 6.4–7.0 ± 0.35 at 25 °C. TEG7 had the most negligible pH of 6.4, while TEG6 had a neutral pH. TEG1, TEG2, TEG3, and TEG4 showed high conductivity, while TEG5, TEG6, TEG7, and TEG8 had low conductivity due to differences in water content. Conductivity values were in the range of 807–915 µS/cm.

#### 2.7.2. Drug Content

The TEG3, TEG4, TEG5 formulations showed fewer drug contents due to phase separation or the settling of drug particles. TEG6 had a high drug content of 96%. Drug content lies in the range of 87–96% and formulations were found to be homogenous.

#### 2.7.3. Spreadability and Extrudability

The spreadability and extrudability of the TELs gel are important parameters for the uniform application of the formulation onto the skin. Spreadability of the formulations was found in the range of 5.18 to 5.65 g/s, whereas the extrudability was in the range of 8.19 to 8.75 g/cm^2^.

#### 2.7.4. Rheological Studies

**Figure 4:**
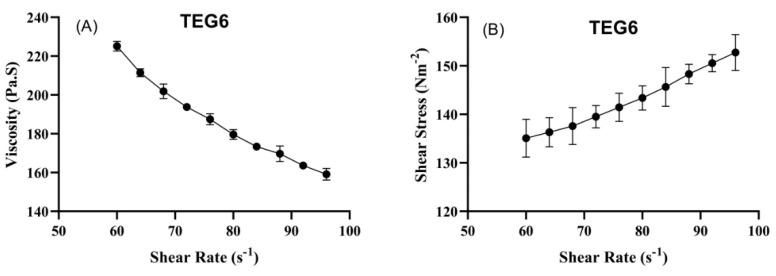
Rheograms of TEG6 between shear rate and viscosity (**A**) and shear rate versus stress (**B**) (Mean ± SD, *n* = 5).

Rheograms of transethosomal gel formulations of TEG6 were plotted between viscosity (*Y*-axis) and shear rate (*X*-axis) to determine the effect of the gel viscosity and shear rate as given in Figure 4. Another direct relation was noted by plotting the graph between shear stress and shear rate in Figure 4. An increase in shear stress resulted in an increased shear rate. The graph showed that an increase in shear rate resulted in a decreased viscosity. An inverse relationship was observed between viscosity and shear rate.

#### 2.7.5. In Vitro and Ex Vivo Drug Release

**Figure 5:**
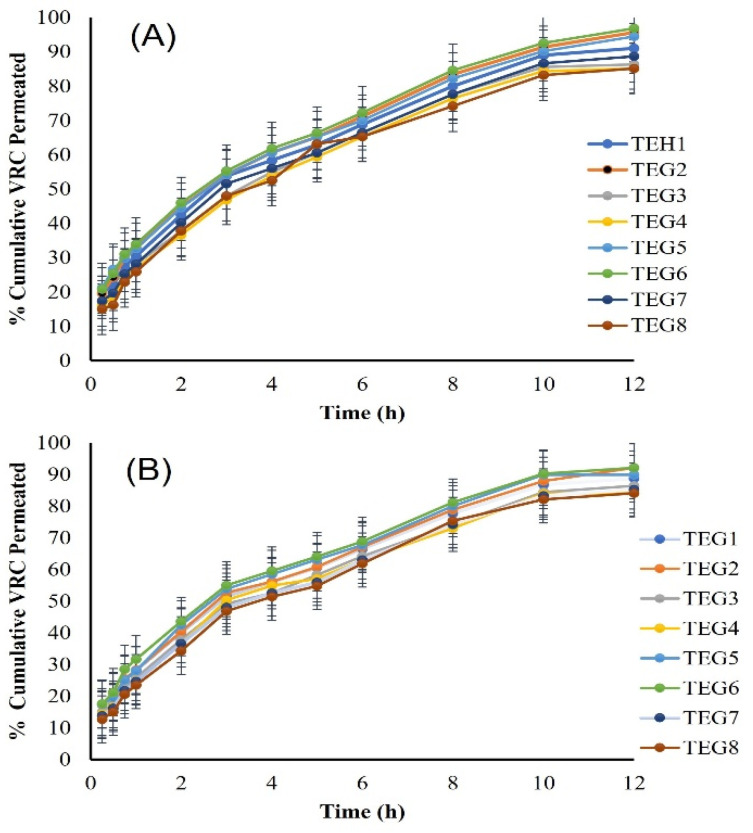
The ex vivo % cumulative voriconazole permeated (**A**) In vitro voriconazole permeated (**B**) of all formulations comparatively (Mean ± SD, *n* = 5).

In vitro release data showed that TEG8, TEG7, TEG4, and TEG3 formulations containing 3% lipoid SPC-3 permeated less via cellophane membrane as compared to TEG1, TEG2, TEG5, and TEG6 formulations. TEG formulations were released up to 92% in 12 h. The ex vivo % VRC that was permeated was also determined as shown by the graph in Figure 5 with a drug release up to 92% in 12 h. The permeation flux (J_max_) at 12 h and the enhancement ratio (ER) of pure VRC and TEG6 formulation are shown in Table 3.

#### 2.7.6. Drug Release Kinetics

Various kinetic models were applied to ascertain the most suitable and best fit model and to interpret the diffusion data in the form of meaningful parameters. The drug release kinetic obeyed the Higuchi model with a Fickian diffusion mechanism as shown in Table 4. Ahmed and co-workers prepared pentoxifylline transferosomes showing a similar result of drug release kinetics [16]. Using the Korsmeyer-Peppas model, the diffusional release exponent (*n*) = 0.5 indicates Fickian diffusion, whereas *n* < 0 < 1.0 is indicative of Non-Fickian (Anomalous) diffusion that shows the release behavior of vesicles following a combination of diffusion as well as erosion. The Higuchi model governs the release of a drug from a permeable matrix where encapsulation of the drug is more than its solubility. It also predicts the drug release in a superior way with more accuracy. Various sustained release products are designed in order to embed the drug in the matrix where the drug dissolves following the penetration of a liquid medium [17,18,19].

#### 2.7.7. Antifungal and Leishmanial Activity

The results of antifungal activity against *Aspergillus fumigatus*, *Aspergillus flavus*, and *Candida albicans* are shown in Table 5. A significant decrease in MIC and MFC was observed in TE6 and TEG6 formulations. The TE6 formulation showed higher antifungal activity as compared to the pure drug and gel formulation. No fungal susceptibility was found for the blank sample. The previously reported values of MIC of VRC against *Aspergillus fumigatus*, *Aspergillus flavus*, and *Candida albicans* are 1.1, 1.7, and 1.6 µg/mL. The previously reported values of MFC of VRC against *Aspergillus fumigatus*, *Aspergillus flavus*, and *Candida albicans* are 4.2, 3.5, and 0.136 µg/mL [20].

**Figure 6:**
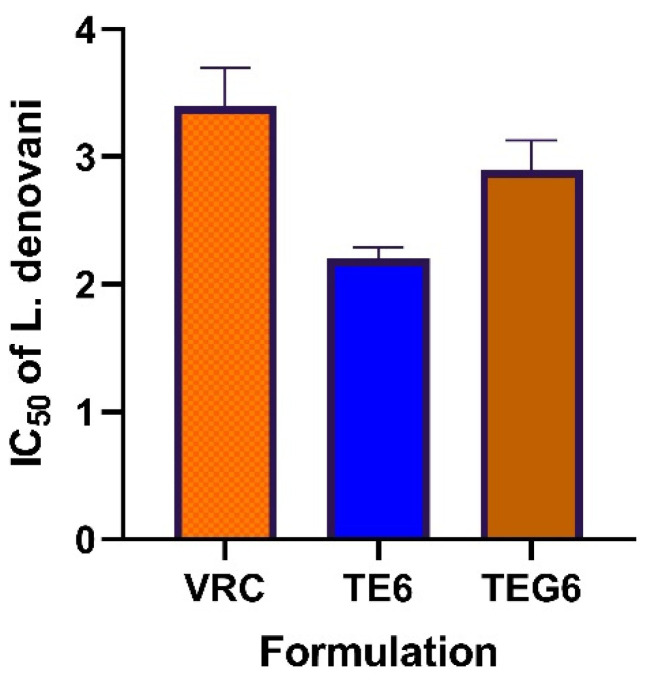
IC_50_ of pure VRC, TEL, and TEG6 against *L. donovani* (Mean ± SD, *n* = 5).

Antileishmanial activity against *L. donovani* is represented in graphical form in Figure 6. The results showed there was a significant increase in the activity of the transethosomal formulation as compared to pure VRC. The activity of VRC loaded TELs formulations may be attributed to their ability to interact with the outer coat of the parasite and deliver the drug.

#### 2.7.8. Molecular Docking Studies

**Figure 7:**
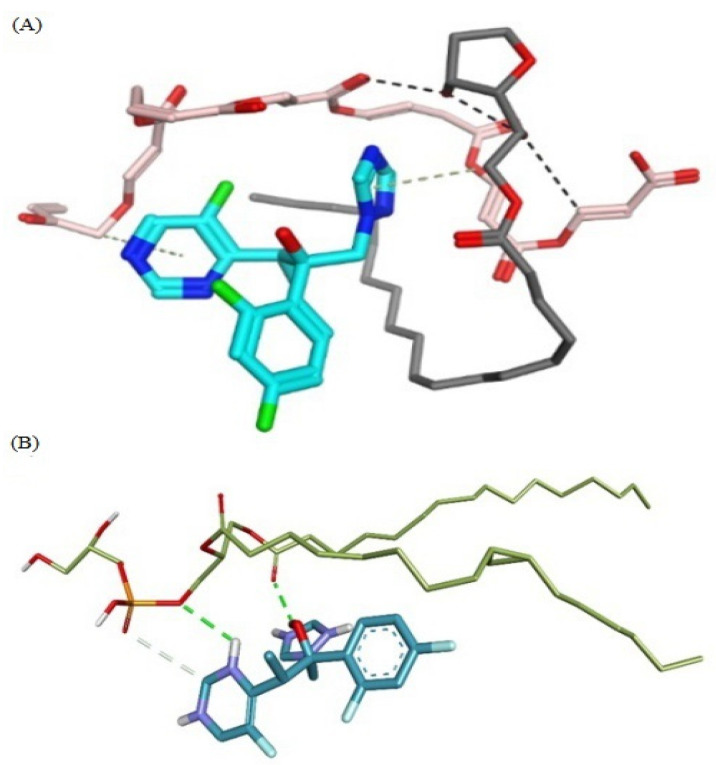
Molecular interactions involved in the stabilization of voriconazole and carbopol. (**A**) The molecular interactions involved in the transethosome stabilization. Voriconazole is presented as cyan carbon sticks while carpobol molecules are presented in pink and grey. (**B**) Green dotted lines show hydrogen bonds, while the light grey dotted lines show π-alkyl interactions.

VRC is presented as cyan carbon sticks, while carbopol molecules are presented in pink and grey colors, respectively. Black dotted lines show polar bonds, while the blue dotted lines show π-hydrophobic interactions. Figure 7 presents the molecular interactions involved in the formation of the stable complex formed between VRC and phospholipids. The complex is stabilized by a number of interactions, such as two-hydrogen bonds between the VRC and phospholipid molecules. One hydrogen bond was formed by triazole moiety and another hydrogen bond was formed by hydroxyl moiety within VRC. This interaction serves as a stability anchor that directs the assembly of the VRC and phospholipids complex, as shown in Figure 7. The triazole ring of the VRC also mediates a π-alkyl interaction with a side chain in the phospholipids. The pyrimidine ring of the compound also extends a similar bonding interaction with carbopol. Green dotted lines show hydrogen bonds, while the light grey dotted lines show π-alkyl interactions.

## 3. Discussion

The present study was designed to investigate the formulation of TELs to incorporate VRC for enhancing antifungal and antileishmanial effects followed by the sustained release of VRC for topical application. Moreover, their nanovesicle size, the presence of a permeation enhancer, and the presence of ethanol and lipid would enable the drug to pass via the stratum corneum, developing the drug availability at their site of action. The physicochemical characterization of VRCT and VTEG was carried out to determine the suitability of the formulation for ex vivo study. The particle size and entrapment efficiency play a decisive role in determining the optimized formulation. It is a well-established fact that the large surface area offered by particle size reduction can significantly enhance bioavailability. The formulation TELs showed an expressively smaller size because of an increased ethanol concentration. The studies reported that particle size has a direct relation with ethanol concentration. By increasing the ethanol concentration, the particle size was also enhanced [7]. Previous research reported that a particle size in the range of 200–300 nm of Paenol-loaded TELs was obtained [21]. The TELs with particles of less than 300 nm can permeate the drug through the stratum corneum. The zeta potential of the formed formulations depends on the type of surfactant/edge activators and ethanol concentration used. High zeta potential indicates highly charged particles in the dispersion medium which prevents aggregation, flocculation, and coagulation. If the zeta potential is low, attraction overcomes repulsion, resulting in coagulation. The zeta potential value of ≥−25 mV or ≤25 mV usually has a high degree of stabilization [22]. The formulation containing Span 60 showed a negative zeta potential, while the formulation containing Tween 80 showed neutral zeta potential, demonstrating that particles are monodispersed [23].

TELs formulation containing VRC, phospholipid, and carbopol 940 mixture showed no chemical interaction among phospholipids, surfactant, and carbopol used with VRC, indicating the chemical stability of VTEG. There was no appearance or disappearance of the characteristic peaks. The quantity of propylene glycol and ethanol used directly affects the EE. Entrapment of the drug was decreased by increasing propylene glycol concentration. Further, an increased concentration of propylene glycol caused the drug to leach out from the vesicles. Conversely, the % EE of the hydrophobic drug was lowered by decreasing the ethanol concentration. Higher ethanol concentration solubilizes the lipid bilayers, leading to leaky TELs with decreased drug loading [24,25]. High ethanol content exhibited the highest % EE. This may be due to the co-solvent effect of ethanol which resulted in the amount of drug accommodated in the aqueous core of vesicles. The ethanol concentration of 30–35% *w*/*w* was chosen because previous reports suggested that 30–35% ethanol is most suitable for achieving a good % EE. Formulation TE2 and TE6 were utterly stable in all aspects of physical stability as well as chemical stability and change in pH [26].

The pH and conductivity of VTEG were in the normal physiological pH range of skin. The values of electrical conductivity were increased continuously due to the presence of excess water. The low conductivity values indicate restricted water mobility, while high values suggest that the system undergoes a structural inversion from water in oil (w/o) to oil in water (o/w) emulsion. These changes have been attributed to the occurrence of a percolation transition [27]. In the percolation threshold model, the conductivity remains low up to a certain critical volume fraction of water (Uc). The w/o droplets surrounded by a continuous oil phase are isolated, thus contributing minimally to the electrical conductance–surfactant effect on the spreadability. The formulation containing Tween 80 showed the least spreadability, while the formulation containing Span 60 showed high spreadability. An inverse relationship was found between viscosity and shear rate [28].

In vitro data showed a strong correlation with ex vivo data. In vitro drug release showed drug release up to 92% in 12 h. The appropriate accuracy of kinetic models was assessed in terms of their correlation coefficient (R^2^). The Higuchi plot was the most appropriate model to elaborate the VTEG as its correlation coefficient (R^2^) values were close to 1 as compared to other models [29]. Initially, no VRC was released from VTEG during this study, showing that VRC was distributed uniformly in the matrix [30]. The n value of VTEG ≤ 0.5. Thus, the mechanism of VRC released from these formulations can be described as Fickian diffusion that occurred by molecular diffusion of the VRC due to a potential chemical gradient [31]. The permeation of the drug was studied via the excised rat skin mainly by a carrier-mediated mechanism. TELs were found to be adequate drug delivery vesicles for penetration into the deeper layer as compared to other deformable vesicles. The ex vivo permeation of VTEG showed a transdermal flux of 22.8 µgcm^−2^ h^−1^ with enhanced efficiency up to 4-fold [16].

The result of antifungal and leishmania activity showed a significant increase in the activity of transethosomal formulation compared to pure VRC. The antileishmanial activity of VRCT formulations may be attributed to their ability to interact with the outer coat of the parasite and deliver the drug. TEG6 depicted better activity than pure VRC as shown in Figure 6 but it was less than TE6 owing to drug release from the gel system. This is due to the more elastic nature of transethosomes. Molecular modeling of carbopol and VRC depicts that the hydrophobic tail of carbopol wraps the polar groups of the VRC. Pi-hydrophobic interactions and hydrogen bonding stabilize the complex. The interaction serves as a bridge that directs the assembly of the complex.

## 4. Materials and Methods

VRC was generously donated by Ferozsons Laboratories, (Pvt) Ltd., KPK, Nowshera, Pakistan. Lipoid S100 and lipoid SPC-3 were gifted from Ludwigshafen AM Rhein, Germany. Propylene glycol, sodium lauryl sulphate, Tween 80, Span 80, cholesterol, ethanol, carbopol 940, and sodium benzoate were purchased from Sigma Aldrich, Germany. All chemicals were of analytical grade and used without further purification.

### 4.1. Preparation of Voriconazole Transethosomes

**Figure 8:**
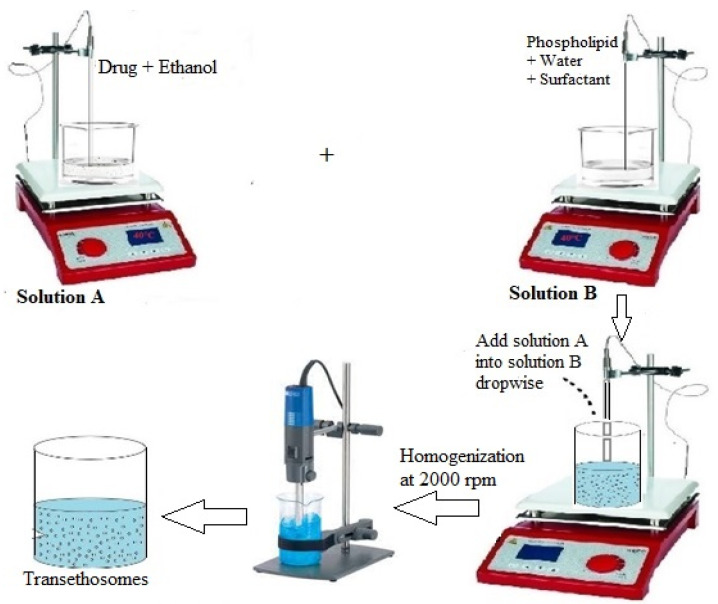
The schematic representation of the cold method.

TELs were prepared by the cold method reported previously using different phospholipids and surfactants with varying concentrations [32]. Phospholipid, cholesterol, surfactant (Twee 80 and Span 80), and drug were added in ethanol, followed by propylene glycol. RO water was added dropwise at 30 °C and stirred vigorously for 5 min at 1200 rpm (Figure 8) by using IKA^®^ C-MAG MS magnetic stirrers (IKA, Staufen, Germany). The suspension was cooled down at a maintained room temperature of 25 °C ± 1. Finally, the TELs suspension was homogenized by using a HG-150 homogenizer (Witeg Labortechnik GmbH, Wertheim, Germany) at 3000 rpm and stored in a refrigerator for further study [32]. The schematic representation of the cold method is shown in Figure 8. The concentration of the different additives and actives used are given in Table 6.

### 4.2. Physiochemical Characterization of Transethosomes

#### 4.2.1. Particle Size, Zeta Potential, and Polydispersity Index

The attraction or repulsion of the charged particles were evaluated by zeta potential. The zeta potential, particle size and polydispersity index were measured using a zeta sizer (ZS-90, Malvern, UK) after diluting suitably with double distilled water [33].

#### 4.2.2. Fourier Transform Infrared Spectroscopy (FT-IR)

Fourier Transform Infrared Spectroscopy (FT-IR) identifies chemical bonds in a molecule by producing an infrared absorption spectrum. The interaction of VRC with TELs was studied by FTIR (Bruker, Tensor 27 series, Rheinstetten, Germany). The FTIR spectra of pure drug, phospholipid, surfactant, and carbopol 940 were recorded on an IR spectrophotometer between 500 and 4000 cm^−1^ with 20 scans [34].

#### 4.2.3. Optical Microscopy

The optical microscopy of TELs was measured by spreading the sample on a glass slide with the help of a glass rod and cover with a coverslip. The samples were observed under a magnification of 40× and 10× [35].

#### 4.2.4. Scanning Electron Microscopy (SEM)

A scanning electron microscope (SEM) produced images of a sample by scanning the surface with a focused beam of electrons. The sample specimen was prepared by mounting a drop of dispersion on-air dried-clear glass slide and sputter- coated with gold on an E 5100 Sputter at an accelerating voltage of 20 kV with a working distance of 15 mm. Then the slide was observed under the scanning electron microscope (JSM-7610FPlus, JEOL, Peabody, MA, USA) [36].

#### 4.2.5. Entrapment Efficiency

The % entrapment efficiency (% EE) was measured by separating unentrapped drug from TELs by centrifugation at 12,000 rpm for 40 min. The quantity of VRC in the sediment was measured by rupturing the vesicles in ethanol. The EE % of VRC in TELs was calculated according to Equation (1).

(1) $$\mathrm{EE}\;(\%)=\frac{\left[{\left(\mathrm{VRC}\right)}_{\mathrm{total}}-{\left(\mathrm{VRC}\right)}_{\mathrm{free}}\right]}{{\left(\mathrm{VRC}\right)}_{\mathrm{total}}}\times 100$$

(VRC)_the total_ represents the weight of the total VRC incorporated in TELs and (VRC)_free_ denotes the weight of unentrapped VRC [37].

#### 4.2.6. Storage Stability of Transethosomes

The VRC concentration in TELs was determined at 30 °C ± 2/65% RH ± 5% RH after 6 mo of storage as per ICH guidelines 2003(Q1A(R2)) by UV Visible spectroscopy. The % of VRC was measured by Equation (2).

(2) $$\mathrm{VRC}\;\mathrm{after}\;\mathrm{storage}\;(\%)=\frac{{\left(\mathrm{VRC}\right)}_{t}}{{\left(\mathrm{VRC}\right)}_{t_{o}}}\times 100$$

where (VRC)_t_ and (VRC)_to_ are the concentration of VRC incorporated in the formulations obtained after 2, 4 and 6 mo of storage at 2–8 °C and at time *t_o_* [8].

### 4.3. Gel Preparation

VRC Transethosomal gel (VTEG) was prepared by using 0.6% carbopol 940 which was dispersed in deionized water and left overnight. After 24 h, the gel was mixed in TELs suspension at 300 rpm, and finally, triethanolamine was added to neutralize the pH [38].

#### 4.3.1. Physical Appearance, pH, and Conductivity

The appearance and clarity of the gel was assessed visually after settling the gels in a transparent container to observe the presence of lumps and phase separation. Formulating an ionizable, poorly water-soluble drug may require an extreme pH value to achieve adequate solubility, stability, and shelf life. The pH of the gel and the conductivity were measured using a pH meter (PHS-550, Hangzhou Lohand Biological Technology Co., Ltd., Hangzhou, China) and a conductivity meter (DDS-22 °C, Hangzhou Lohand Biological Technology Co., Ltd., Hangzhou, China) at 25 °C.

#### 4.3.2. Spreadability and Extrudability

Spreadability was measured by taking two glass slides with the middle point marked on fixed slides. The sample was applied on a marked point. The second glass slide was placed on fixed slides. Standard weight was placed on moveable slides. Spreadability was measured by using Equation (3).

S = M/t
(3)

‘S’ represents spreadability in g/s, ‘M’ denotes the weight of gel in grams, and ‘t’ is the time taken by the gel in seconds.

Extrudability was measured by extruding an empirical quantity of the drug from the aluminum tube by applying constant weight from 0.5 cm strip of gel in time ‘t’ [39].

#### 4.3.3. % Drug Content

The % drug content of the formulations was analyzed by a UV/VIS spectrophotometer (Perkin Elmer lambda 25, Germany) at 255 nm by dissolving 1 g of gel in ethanol. The sample was diluted with ethanol and absorbance was noted at 255 nm [40].

#### 4.3.4. Rheological Studies

The rheological behavior of VTEG was measured using a Rheometer (DV-III ultra-Rheometer, Brookfield, Germany) at 25 °C. The viscosity behavior in relation to applied stress was noted [41].

#### 4.3.5. In Vitro and Ex Vivo Permeation Study

The permeation of the drug was determined using a Franz diffusion cell (PERME GEAR, Hellertown, PA, USA) in a phosphate buffer of pH 7.4. The receptor compartment was filled with buffer pH 7.4 buffer and equilibrium was maintained for 30 min at 37 ± 2 °C with stirring at 100 rpm. A drug sample was applied on the cellophane membrane that was mounted on the receptor compartment. A 1 mL sample was taken periodically up to 12 h and replaced by an equal quantity of buffer. Drug concentration was determined by using a UV/VIS Spectrophotometer (Perkin Elmer Lambda 25). For the ex vivo permeation study, freshly prepared albino rat skin was used with an area of 1.76 cm^2^. The % release of the drug was calculated using Equation (4) [42]. The flux and coefficient of the permeability were calculated by using Equations (5) and (6).

Concentration = C_i_ × D_f_ × V_r_ (4)

(5) $$\mathrm{Flux}\;(J)=\frac{Q\;}{t\times S}$$

(6) $$\mathrm{Kp}=\frac{J}{\mathrm{Cd}}$$

C_i_ is the concentration of drug calculated from the standard curve; D_f_ is the dilution factor; V_r_ is the volume of the receptor compartment. Q denotes the quantity of the drug permeated through the skin. The surface is S and time is ‘t’. Cd represents drug concentration in the donor compartment.

#### 4.3.6. Drug Release Kinetics

Zero-order, first-order, Higuchi, and Korsmeyer-Peppas release models were applied for elaborating the mechanism of the drug released and calculated by Equations (7)–(10), respectively.

(7) $$F_{r}=k_{0}t$$

(8) $$\ln\;(1-F)=-K_{f}t$$

(9) $$F_{i}=K_{H\;}t_{½}$$

(10) $$\frac{\mathrm{Wt}}{W}=K_{p}t_{n}$$

F_i_ and F_r_ are initial fractions and released a fraction of the drug in time ‘t’. K_0_, K_f_, K_H_, and K_P_ represent the zero order, first order, Higuchi and Korsmeyer-Peppas release constants. W, Wt and n denote the mass of water absorbed at equilibrium, the water absorbed in the time (t), and n denotes the release exponent.

#### 4.3.7. Antileishmanial Activity

In vitro *leishmanial* activity of TELs loaded VRC, pure VRC (C_d_), a blank sample (B_f_), and VTEG were performed against *Leishmania* promastigotes. The 96 Well microtiter plate assay method was used for the antileishmanial activity. The stock solution was prepared by adding 1 mg VRC equivalent VTEG sample in 50 µL of dimethyl sulfoxide, and the volume was made up to 1000 µL by RPMI-1640 media. The organism was grown in RPMI-1640 liquid media containing 10% serum of fetal protein (bovine). The parasites were centrifuged at 2000 rpm for 10 min at log phase. The supernatant was discarded and fresh media was used until the density reached 100,0000 cells per mL. It was added to wells in 100 µL and 180 µL such that only the first column received 180 µL and the last two rows acted as a positive and negative control. First, 20 µL of stock solution was added and mixed. This order must be followed to make serial dilutions. This was incubated at 25 °C for four days in a dark place and examined under the microscope using a Neubauer Chamber. *Leishmania* promastigotes were used as a test organism. Leshmanial parasite growth inhibition was determined using a luciferase enzyme by measuring the relative luminescence unit (RLU) [43].

#### 4.3.8. Antifungal Activity

The antifungal activity of VRC was determined against *Candida albicans, Aspergillus flavus,* and *Aspergillus fumigatus* with *ATCC 10231*, *ATCC 16883* and *ATCC 96918* numbers, respectively. The sample was dissolved in RPMI 1640 medium. The pH was adjusted to 7.0 by 0.165 M morpholine propane sulfonic acid. After mixing, a 100 mL sample was taken with a concentration of 0.15 mg/mL for VRC loaded TELs and pure VRC solution in phosphate buffer saline. The growth of yeast inoculum was carried out at 35 °C for 24 h on the Sabouraud dextrose agar plates to a concentration range of 0.6–2.6 µg/mL. Inoculum were added to microdilution plates containing 0.1 µg/mL to 10 µg/mL of drug solution and incubated at 30 ± 5 °C for 1–2 days. Minimum inhibitory concentration (MIC_90_) and minimum fungicidal concentration (MFC) were measured. Optical density was measured at 570 nm using a microplate reader [44,45].

#### 4.3.9. Molecular Docking Studies

The compounds were sketched using the builder module in the Molecular Operating Environment (MOE v 2018.0101) software (Chemical Computing Group ULC, 1010 Sherbrooke St. West, Suite #910, Montreal, QC, Canada, H3A 2R7, 2018). The structure of VRC was obtained in the smile format from the drug bank web server. Additionally, the structure of phospholipid was obtained from PubChem. The partial atomic charges were calculated followed by energy minimization according to a steepest-descent protocol using the Merck Molecular Force Field (MMFF94X) in MOE with a Root Mean Square gradient of 0.01 Å. All the calculations were performed according to the stoichiometric calculations in the present study at a constant temperature (310 K) in a vacuum. The resulting structures were analyzed visually. All the graphics were rendered using MOE [46].

#### 4.3.10. Statistical Analysis

The data was statistically analyzed to determine their significance level. Data are presented as mean ± Standard deviation (SD) from at least five samples unless mentioned. The data was evaluated using analysis of variance (ANOVA) followed by other suitable statistical parameters as required. Statistical significance was designed as *p* < 0.05.

## 5. Conclusions

The present study elaborates the fabrication of transethosomes of VRC followed by their incorporation into a gel for antifungal and antileishmanial applications. Transethosomes were characterized by zeta potential, entrapment efficiency, particle size, polydispersity index (PDI), optical microscopy, scanning electron microscopy (SEM), Fourier-transform infrared spectroscopy (FTIR), drug release studies, and stability evaluations. In addition, the formulated VTEG was characterized for various physicochemical aspects, drug release studies, molecular docking, antifungal, and antileishmanial activities. The results demonstrated that VTEG significantly enhanced the antifungal activity against susceptible strains. The enhanced antileishmanial activity was also evidenced. Finally, it can be concluded that the developed VTEG can be highly beneficial in treating topical fungal infections besides its efficacy for leishmanial parasites and can result in potential benefits in the therapy of various fungal infections.

## Figures and Tables

**Table 1:** Particle size, zeta potential, and PDI of nanovesicles (Mean ± SD, *n* = 5).

Sample code	Zeta Potential (mV)	Particle Size (nm)	PDI *
TE1	32 ± 0.21	133.9 ± 010	0.41 ± 0.04
TE2	−25.2 ± 0.01	229.8 ± 0.06	0.45 ± 0.11
TE3	29 ± 0.11	183.4 ± 0.11	0.46 ± 0.04
TE4	−27.5 ± 0.00	135.5 ± 0.03	0.45 ± 0.09
TE5	25 ± 0.13	302.1 ± 0.06	0.48 ± 0.02
TE6	−26.5 ± 0.01	228.2 ± 0.13	0.45 ± 0.05
TE7	27 ± 0.12	336.3 ± 0.10	0.46 ± 0.02
TE8	−28.3 ± 0.24	342.5 ± 0.11	0.49 ± 0.06

* PDI: Polydispersity Index.

**Table 2:** Physio-chemical behavior of transethosomes after six months of stability (mean ± SD, *n* = 5).

Formulation Code	TE1	TE2	TE3	TE4	TE5	TE6	TE7	TE8
pH	6.20 ± 0.05	6.60 ± 0.13	6.1 ± 0.11	6.2 ± 0.02	6.5 ± 0.07	6.8 ± 0.19	6.3 ± 0.5	6.7 ± 0.07
% EE	81.2 ± 0.13	85.5 ± 0.17	76.1 ± 0.34	72.7 ± 0.43	83.4 ± 0.22	86.3 ± 0.81	73.2 ± 0.32	75.1 ± 1.31
Particle size (nm)	135.6 ± 0.11	228.3 ± 0.25	260.3 ± 0.22	235.7 ± 0.09	304 ± 0.13	331 ± 1.12	476 ± 0.19	488 ± 0.75
PDI	0.40 ± 0.07	0.44 ± 0.23	0.48 ± 0.09	0.49 ± 0.37	0.45 ± 0.35	0.47 ± 0.09	0.55 ± 034	0.51 ± 0.62

**Table 3:** The permeation flux (Jmax) at 12 h and the enhancement ratio (ER) of pure VRC and TEG6 formulation.

Sample Code	J (µgcm^−2^ h^−1^)	K_p_ (cmh^−1^)	Enhancement Ratio (ER)
Voriconazole	4.9	0.57	4.6
TEG6	22.8	2.6	

**Table 4:** Drug release kinetics of VRC.

Sample Code	Zero-Order Model		1st Order Model		Higuchi Model		Korsmeyer-Peppas Model		
	*k*_0_	*R* ^2^	*k*_1_	*R* ^2^	KH	*R* ^2^	KKP	*n*	*R* ^2^
TEG1	9.74	0.44	0.24	0.90	28.23	0.98	31.9	0.44	0.99
TEG2	10.12	0.43	0.26	0.90	29.35	0.98	33.46	0.43	0.99
TEG3	9.29	0.53	0.21	0.92	26.79	0.98	28.96	0.46	0.99
TEG4	9.14	0.57	0.19	0.93	26.30	0.99	27.82	0.47	0.99
TEG5	10.02	0.35	0.25	0.87	29.12	0.97	34.07	0.41	0.99
TEG6	10.28	0.38	0.27	0.89	29.84	0.97	34.63	0.42	0.99
TEG7	9.44	0.52	0.22	0.92	27.23	0.98	29.59	0.45	0.99
TEG8	9.10	0.58	0.19	0.94	26.20	0.99	27.47	0.47	0.99

**Table 5:** Antifungal activity MIC and MFC value in µg/mL (Mean ± SD, *n* = 5).

Species name	Formulation	MIC (µg/mL)	MFC (µg/mL)
*Aspergillus fumigatus*	VRC	1.1 ± 0.05	4.2 ± 0.02
	TEG6	0.80 ± 0.11	3.2 ± 0.04
	TE6	0.75 ± 0.07	3 ± 0.12
	BF	____	____
*Aspergillus flavus*	VRC	1.7 ± 0.03	3.5 ± 0.08
	TEG6	1.4 ± 0.12	3.1 ± 0.012
	TE6	1.5 ± 0.04	3.29 ± 0.13
	BF	____	____
*Candida albicans*	VRC	1.6 ± 0.05	0.136 ± 0.11
	TEG6	0.055 ± 0.013	0.133 ± 0.01
	TE6	0.053 ± 0.01	0.129 ± 0.04
	BF	____	____

**Table 6:** Composition of transethosomes of voriconazole.

Ingredients (% *w*/*w*)	TE1	TE2	TE3	TE4	TE5	TE6	TE7	TE8
Lipoid S100	3	3	---	---	3	3	---	---
Lipoid SPC-3	---	---	2	2	---	---	1	1
Cholesterol	0.5	0.5	0.5	0.5	0.5	0.5	0.5	0.5
Propylene glycol	1	1	1	1	1	1	1	1
Voriconazole	1	1	1	1	1	1	1	1
Ethanol	30	30	30	30	35	35	35	35
Tween 80	0.54	---	0.54	---	0.54	---	0.54	---
Span 80	---	0.54		0.54	---	0.54	---	0.54
Sodium benzoate	0.02	0.02	0.02	0.02	0.02	0.02	0.02	0.02

## Data Availability

The data presented in this study are available from the authors upon reasonable request.

## Sample Availability

Samples of the compounds (voriconazole-loaded transethosomal gel) are available from the authors.
